# Different evolutionary patterns of SNPs between domains and unassigned regions in human protein-coding sequences

**DOI:** 10.1007/s00438-016-1170-7

**Published:** 2016-01-30

**Authors:** Erli Pang, Xiaomei Wu, Kui Lin

**Affiliations:** MOE Key Laboratory for Biodiversity Science and Ecological Engineering, College of Life Sciences, Beijing Normal University, Beijing, 100875 China; College of Life and Environmental Sciences, Hangzhou Normal University, Hangzhou, 310036 China

**Keywords:** Human genome, Protein-coding sequence, Protein domain, SNPs, Natural selection

## Abstract

Protein evolution plays an important role in the evolution of each genome. Because of their functional nature, in general, most of their parts or sites are differently constrained selectively, particularly by purifying selection. Most previous studies on protein evolution considered individual proteins in their entirety or compared protein-coding sequences with non-coding sequences. Less attention has been paid to the evolution of different parts within each protein of a given genome. To this end, based on PfamA annotation of all human proteins, each protein sequence can be split into two parts: domains or unassigned regions. Using this rationale, single nucleotide polymorphisms (SNPs) in protein-coding sequences from the 1000 Genomes Project were mapped according to two classifications: SNPs occurring within protein domains and those within unassigned regions. With these classifications, we found: the density of synonymous SNPs within domains is significantly greater than that of synonymous SNPs within unassigned regions; however, the density of non-synonymous SNPs shows the opposite pattern. We also found there are signatures of purifying selection on both the domain and unassigned regions. Furthermore, the selective strength on domains is significantly greater than that on unassigned regions. In addition, among all of the human protein sequences, there are 117 PfamA domains in which no SNPs are found. Our results highlight an important aspect of protein domains and may contribute to our understanding of protein evolution.

**Electronic supplementary material:**

The online version of this article (doi:10.1007/s00438-016-1170-7) contains supplementary material, which is available to authorized users.

## Introduction

Studying protein evolution is crucial for understanding the evolution of speciation and adaptation, senescence and human genetic disease (Pál et al. 2006). At the sequence level, protein evolution occurs primarily through two processes: the random production of DNA mutations and the fixation of new variations in populations, which is constrained simultaneously by selection and the population size. Single nucleotide polymorphisms (SNPs) are abundant within populations and represent a major form of genomic variation. SNPs are widely exploited as genetic markers for phenotypic differences (Sachidanandam et al. 2001; Suh and Vijg 2005). As a result, SNPs in protein-coding sequences are of particular interest and have been explored extensively in many organisms.

In the pre-whole-genome era, researchers focused on SNPs in different types of proteins. For example, while investigating 182 housekeeping and 148 tissue-specific genes in humans Zhang and Li (2005) found no evidence of positive selection for either gene class, while Cohuet et al. (2008) studied 72 immune related genes and 37 randomly chosen genes in *Anopheles gambiae* and detected similar patterns and rates of molecular evolution in both categories. The growing numbers of published population genomics studies has increased the availability of genome-scale SNP data sets (Liti et al. 2009; Schacherer et al. 2009; Abecasis et al. 2010; Abecasis et al. 2012), which makes it possible to survey detailed selections from complete genomes. Using more than 11,000 human protein-coding genes, Bustamante et al. (2005) observed that selection acting on genes participating in different biological process and molecular functions varies greatly. In *Drosophila simulans*, Begun et al. (2007) discovered that adaptive protein evolution is common, while a genome-wide survey of SNPs in *Saccharomyces paradoxus*, Vishnoi et al. (2011) confirmed that purifying selection within the *S. paradoxus* lineage is ongoing.

In general, there are many types of evolutionary forces at play during the course of genome sequence evolution; thus, they should impose different and/or subtle constraints on different classes of genomic sequences. For example, constraints on coding-gene sequence, mainly by purifying selection, are stronger than those on most, if not all, non-coding sequences. However, this does not imply that there are uniform constraints across all sequences within a class, and much evidence shows that most sites are differently constrained even within a segment of sequence that constitutes a functional unit (Nielsen 2005; Tian et al. 2008; Koonin and Wolf 2010). For example, Mu et al. (2011) analyzed non-coding elements that were classified into three categories and showed that each had a very distinct variation profile. Most protein sequences are composed of domains, which usually convey distinct functions (Bateman et al. 2002; Koonin et al. 2002; Ponting and Russell 2002). Recently, Yates and Sternberg (2013) analyzed human non-synonymous SNPs to identify disease-resistant and disease-susceptible domains and proteins. In the present study, we explored the distribution of SNPs located in human protein-coding genes (cSNPs) and sought to determine whether there is any significant difference between the distribution patterns of cSNPs when each protein sequence is divided into two groups: the first of which contains PfamA-classified domains, whereas the second group contains unassigned regions (i.e., for each protein, those sequences not annotated by the PfamA database). The SNP dataset was parsed from the newly available genetic variation from 1092 human genomes (Abecasis et al. 2012) according to the GENCODE annotation of protein-coding genes (version 7) (Harrow et al. 2006), whereas the PfamA domain annotation is from the Pfam database, version 27.0. Based on this information, we surveyed the following: (1) the strength of selection acting on SNPs, partitioned into SNPs in domains (doSNPs) and SNPs in unassigned regions (unSNPs); and (2) the density of non-synonymous, and synonymous SNPs, classified into two types. We found that there are significantly different evolutionary patterns between domains and unassigned regions in the human genome. In addition, we found that there are 117 domains for which no SNP has been identified. Our results provide new insight into the existing pool of knowledge regarding the evolution and function of human proteins.

## Materials and methods

### Overview of our approach

Our analysis is based on a whole-genome set of genetic variations from 1092 human genomes. It involves five steps: (1) mapping SNPs on protein coding sequences; (2) classifying SNPs into non-synonymous (nsSNPs) and synonymous variations (sSNPs); (3) annotating the proteins with PfamA domains; (4) dividing the SNPs into doSNPs and unSNPs; and (5) obtaining the fixed variations in human. We provide the details of data sources and analysis methods for all.

### Data sources

In this study, we mainly used six types of data: genome sequence, genome annotation, genome-wide variations from human populations, principal splice isoforms for human genes (Manuel Rodriguez et al. 2015), PfamA domains and the Enredo-Pecan-Ortheus (EPO) primate alignments (Hubbard et al. 2009).

The genome-wide set of genetic variations from 1092 human genomes (Abecasis et al. 2012) was downloaded from the 1000 Genomes Project (http://www.1000genomes.org/). The human genome sequence used was based on the February 2009 *Homo sapiens* assembly, GRCh37, downloaded from Ensembl (Flicek et al. 2013) (http://asia.ensembl.org/index.html). Meanwhile, the ancestral sequences with high-confidence calls for *H. sapiens* (GRCh37) were retrieved from the 1000 Genomes Project (ftp://ftp.1000genomes.ebi.ac.uk/vol1/ftp/phase1/analysis_results/supporting/ancestral_alignments/). The models of the protein-coding genes were retrieved from version 7 of the GENCODE project (December 2010 freeze), whose aim is to annotate all evidence-based gene features in the human genome (Harrow et al. 2006) (http://www.gencodegenes.org/). The 6 way EPO primate alignments were downloaded from Ensembl (ftp://ftp.ensembl.org/pub/release-71/emf/ensembl-compara/epo_6_primate). Based on these datasets, the protein-coding sequences and their related SNPs were extracted using our Perl script. For those genes with multiple transcripts, the principal isoform from APPRIS database (http://appris.bioinfo.cnio.es/#/downloads) was selected; in total, 20,571 protein-coding sequences and their corresponding protein-coding sequences were used for the following analysis.

### Domain assignment

We used Pfam database (http://pfam.sanger.ac.uk/) (Punta et al. 2012) (Pfam27.0 release, March 2013), which contains 14,831 domains. The proteins were assigned domains using pfam_scan.pl downloaded from Pfam (*E* value ≤10^−3^). After this domain annotation, each protein was partitioned into two parts: the domain regions mapped by any Pfam domain and the unassigned regions for the remainder unmapped sequences. All the cSNPs were also divided into two groups: doSNPs if they were within the domain regions and unSNPs when they were not.

### Fixed divergence

Divergence information of protein-coding sequences between humans and their ancestors was identified using our Perl script. The ancestral sequences were from the 1000 Genomes Project, we only used high-confidence call: ancestral state was supported by the other two sequences. A mutation is considered as a fixed divergence if the corresponding site is not polymorphic in human populations and not missing chimp information in the 6 way EPO primate alignments as well.

### Calculation of the direction of selection

Direction of selection (DoS) provides a statistic to estimate the patterns of selection based on numbers of non-synonymous polymorphism (*P*_n_), synonymous polymorphism (*P*_s_), non-synonymous substitutions (*D*_n_), and synonymous institutions (*D*_s_) (Stoletzki and Eyre-Walker 2011). DoS was defined as *D*_n_/(*D*_n_ + *D*_s_) − *P*_n_/(*P*_n_ + *P*_s_).

### Inference of the strength of purifying selection acting on domains and unassigned regions

The method proposed by Eyre-Walker et al. (2006) was used to infer the strength of purifying selection. The software was downloaded from http://www.lifesci.sussex.ac.uk/home/Adam_Eyre-Walker/Website/Software.html.

### The density of sSNPs (or nsSNPs)

The density of sSNPs (or nsSNPs) is the number of synonymous (or non-synonymous) polymorphisms per synonymous (or non-synonymous) site. We counted the number of synonymous (or non-synonymous) SNPs and the number of synonymous (or non-synonymous) sites for domains and unassigned regions, respectively. The odd ration of them is defined as the density of sSNPs (or nsSNP).

### Assessment of differences in amino acid compositions between domains and unassigned regions

For the proteins, we counted the number of each type of amino acids (total 20 types of amino acids) in domains and unassigned regions, respectively. We considered the result to indicate significant differences in amino acid composition between domains and unassigned regions if the 20 types of amino acids had significant difference according to Chi square tests (*p* value <0.05).

### Assessment of codon usage bias

To assess the codon usage bias, we calculated effective number of codons (ENC) with CodonW (http://codonw.sourceforge.net/). The reported value of ENC is always between 20 (when only one codon is effectively used for each amino acid) and 61 (when codons are used randomly). In this work, genes have no significant codon bias when the ENC value is more than 50.

### Randomization process

A randomization process was used to measure whether the number of domains without any SNPs is statistically significant. First, we randomly assigned all their N observed SNPs to positions in the human proteins. This randomization process was repeated 1000 times. Then we counted how many times the number of domains without SNPs is greater or equal than 117, and how many times the average occurrences of domains without SNPs is higher or equal than the one observed for the origin 117 domains. Finally, we can obtain empirical *p*-values, which are the ratios of the times that the value of domains without SNPs is greater or equal that the one observed for the origin 117 domains.

### Statistical tests

Fisher’s exact test was used to test difference of the density of SNPs. The difference of amino acid compositions was tested by Chi square test. Mann–Whitney test was used to test the difference of lengths of two groups of domains. Spearman’s rank test was used to test correlation between paired samples. All statistical tests were performed using the R statistical package.

## Results

### Classification of SNPs within human protein-coding sequences

Using the human genome based on the GRCh37 assembly and genome annotation version 7, 20,571 protein-coding genes were identified (excluding genes on the Y chromosome and in the mitochondrial genome). Because 92–94 % of the genes undergo alternative splicing (Wang et al. 2008), we extracted the principal splice isoform for each protein-coding gene basing on APPRIS database, which designated one of the isoforms as the principal isoform integrating protein structural information, functionally important residues, conservation of function domains and evidence of cross-species conservation (Manuel Rodriguez et al. 2015). By mapping the SNPs from 1092 human genomes (Abecasis et al. 2012) onto these genes, we identified 19,909 genes with cSNPs. We observed 492,826 polymorphic nucleotides, of which 291,485 altered the amino acid sequences and 201,341 were synonymous.

Mapping the 14,831 domain profiles in Pfam27.0 (Punta et al. 2012) onto human protein sequences enabled 5426 PfamA domains to be assigned in the proteins. Therefore, each of the protein sequences was simply divided into two parts: domains (annotated by PfamA domains) and unassigned regions (the remainder of the sequence). In total, there are 14,557,293 nucleotides in domains and 18,411,513 nucleotides in unassigned regions. Thus, the respective cSNPs were also separated into two types: doSNPs and unSNPs. Each type of SNP was partitioned according to minor allele frequency (MAF), as denoted by rare (MAF <0.5 %), low (0.5 % ≤ MAF ≤ 5 %) and common (MAF >5 %) SNPs (Table 1).

**Table 1:** Summary of polymorphisms and divergence

	Rare (MAF <0.5 %)	Low (0.5 % ≤ MAF ≤ 5 %)	Common (MAF >5 %)
Polymorphism 19,909 genes			
Non-synonymous SNPs			
Domains	101,551	13,172	6965
Unassigned regions	135,585	22,134	12,078
Synonymous SNPs			
Domains	68,916	15,092	10,063
Unassigned regions	77,722	18,160	11,388
Divergence (fixed) 15,649 genes			
Non-synonymous changes			
Domains	10,153		
Unassigned regions	21,810		
Synonymous changes			
Domains	18,988		
Unassigned regions	23,626		

Using high-quality ancestral sequences filtering the sites chimp missing, we identified 15,649 genes with fixed mutations. In all, we found 74,577 fixed changes derived from humans; 31,963 were non-synonymous and 42,614 were synonymous. These changes were divided into four types (Table 1).

### Stronger purifying selection pressure on domains than on unassigned regions

Based on PfamA, all SNPs were classified as either doSNPs or unSNPs. First, we used DoS to measure the relative roles of purifying and positive selection acting on domains and unassigned regions (see “Materials and methods”). DoS is calculated using the numbers of non-synonymous and synonymous fixed diversities and polymorphisms. The data used to calculate DoS was shown in Table 2. The DoS were −0.21 and −0.13 for domains and unassigned regions, respectively. This indicates that domains and unassigned regions are under purifying selection.

**Table 2:** Direction of selections for domain and unassigned regions

Type of regions in 15,649 genes	Non-synonymous SNPs	Synonymous SNPs
Domain regions		
Fixed divergence	10,153	18,988
Polymorphisms	104,956	81,593
Direction of selection	−0.21	
Unassigned regions		
Fixed divergence	21,810	23,626
Polymorphisms	153,022	96,960
Direction of selection	−0.13	

Direction of selection: *D*_n_/(*D*_n_ + *D*_s_) − *P*_n_/(*P*_n_ + *P*_s_)

**Fig. 1:**
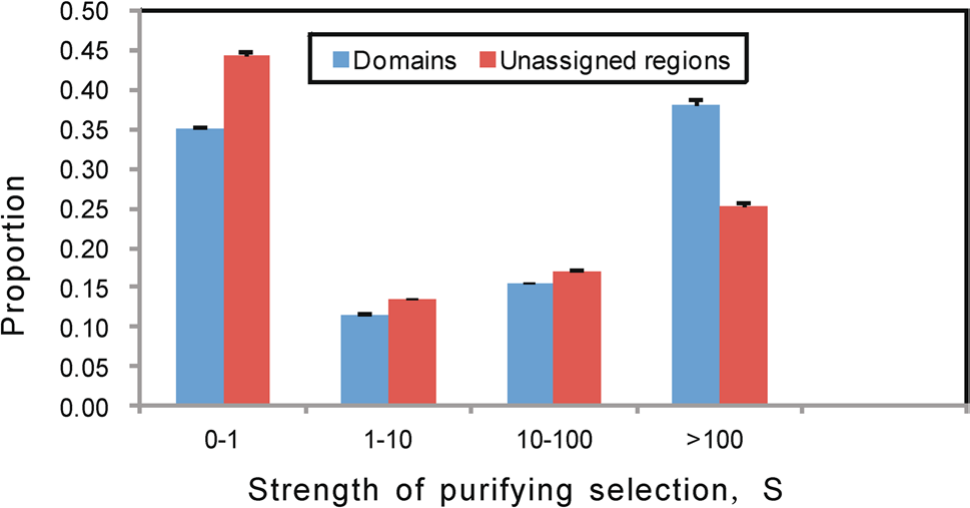
Distribution of fitness of non-synonymous in domains and unassigned regions. *Error bars* denote SE around estimated proportions

Then, we want to know the strength of selection acting on domains and unassigned regions. Because DoS can’t be used to quantify the strength of purifying selection, we used the likelihood-based method of Eyre-Walker et al. (2006) (see “Materials and methods”) to infer the gamma distribution of fitness effects. The advantage of this method is that it can control demographic effects. The sharp parameters of domains and unassigned regions were 0.13 (0.12, 0.13) and 0.12 (0.11, 0.12), respectively. The mean strength of purifying selection acting on domains and unassigned regions were 1.86e+3 (1.69e+3, 2.1e+3) and 4.56e+2 (4.23e+2, 5.23e+2), respectively. The proportion of mutations falling within four categories of *S* values reflects different strengths of selection on both domains and unassigned regions (Fig. 1). We found domains exhibiting the lower fraction of mutations with |*S*| < 1 (35 %) than that of unassigned regions (44 %). This suggests that purifying selection on domains is stronger than that on unassigned regions.

### Greater constraint on the synonymous SNPs in unassigned regions than on those in domains

There is another question of whether there is any difference between domains and unassigned regions in human protein-coding sequences for non-synonymous/synonymous SNPs. In order to answer the question, the cSNPs were partitioned into four types: non-synonymous doSNPs, non-synonymous unSNPs, synonymous doSNPs, and synonymous unSNPs basing on all SNPs being classified as either doSNPs or unSNPs. We then calculated the density for each of them (see “Materials and methods” for details).

**Fig. 2:**
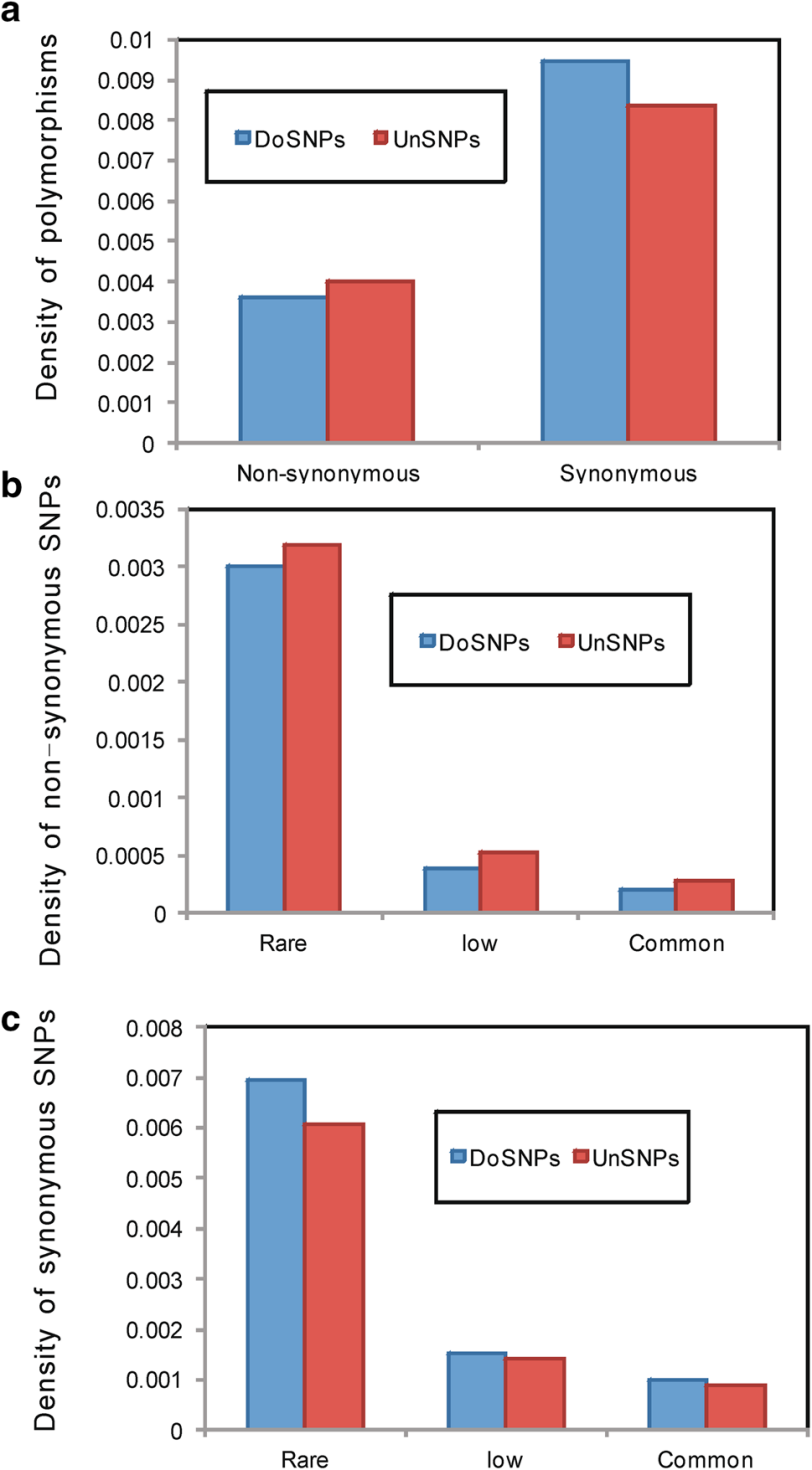
Density of SNPs in domains and unassigned regions. **a** Density of non-synonymous and synonymous SNPs (Fisher’s exact test: *ρ* = 0.90, *p* < 2.2 × 10^−16^ and *ρ* = 1.14, *p* < 2.2 × 10^−16^, respectively). **b** Density of different MAF non-synonymous SNPs (Fisher’s exact test, *ρ* = 0.94, *p* < 2.2 × 10^−16^, *ρ* = 0.75, *p* < 2.2 × 10^−16^ and *ρ* = 0.73, *p* < 2.2 × 10^−16^, respectively). **c** Density of different MAF synonymous SNPs (Fisher’s exact test, *ρ* = 1.14, *p* < 2.2 × 10^−16^, *ρ* = 1.07, *p* < 3.27 × 10^−10^, and *ρ* = 1.14, *p* < 2.2 × 10^−16^, respectively)

First, we observed the non-synonymous SNPs. As shown in Fig. 2a the density of non-synonymous doSNPs was significantly lower (Fisher’s exact test: *ρ* = 0.90, *p* < 2.2 × 10^−16^) than that of unSNPs. We further analyzed the densities of different MAF non-synonymous SNPs. For different MAF non-synonymous doSNPs, the densities were all significantly lower than those of non-synonymous unSNPs (Fisher’s exact test, *ρ* = 0.94, *p* < 2.2 × 10^−16^, *ρ* = 0.75, *p* < 2.2 × 10^−16^ and *ρ* = 0.73, *p* < 2.2 × 10^−16^, respectively for rare, low and common SNPs, Fig. 2b). This is consistent with our intuition and suggests that there are greater constraints on the non-synonymous doSNPs than on the non-synonymous unSNPs.

Next, we surveyed the synonymous SNPs. As described in Fig. 2a, there was a different pattern with that of the non-synonymous SNPs. The density of synonymous doSNPs was significantly greater (Fisher’s exact test: *ρ* = 1.13, *p* < 2.2 × 10^−16^) than that of unSNPs. We further analyzed the densities of different MAF synonymous SNPs and found that the densities of different MAF synonymous doSNPs were all significantly greater than those of synonymous unSNPs (Fisher’s exact test, *ρ* = 1.14, *p* < 2.2 × 10^−16^, *ρ* = 1.07, *p* < 3.27 × 10^−10^, and *ρ* = 1.14, *p* < 2.2 × 10^−16^, respectively for rare, low and common SNPs, Fig. 2c).

We recognized that these results could stem from the different amino acid compositions between the two types of sequences. To control for this, we did not consider genes with significant differences in amino acid compositions of two parts (Chi square tests, *p* < 0.05) (see “Materials and methods”). After filtering, 5480 proteins remained, at which point we repeated the analysis and found similar patterns with the whole protein set (Fisher’s exact test, *ρ* = 0.86, *p* < 2.2 × 10^−16^ and *ρ* = 1.09, *p* < 2.2 × 10^−16^, respectively for non-synonymous SNPs and synonymous SNPs) (Supplementary Figure S1).

The codon usage bias of proteins might affect on our results. To remove the potential influence of codon usage bias, we excluded proteins with ENC less than or equal to 50 (see “Materials and methods”). We obtained 9768 proteins in which codon usage has no bias. We analyzed the protein set, and the patterns were also consistent (Fisher’s exact test, *ρ* = 0.88, *p* < 2.2 × 10^−16^ and *ρ* = 1.08, *p* < 2.2 × 10^−16^, respectively for non-synonymous SNPs and synonymous SNPs) (Supplementary Figure S2).

These results implied that our observation was affected by many factors. Synonymous mutations have been found to be the causes and consequences of codon bias (Plotkin and Kudla 2010; Weatheritt and Babu 2013) and to affect protein translation and folding (Kimchi-Sarfaty et al. 2007; Poliakov et al. 2014). Recently, Lawrie et al. found strong purifying selection at synonymous sites in *Drosophila melanogaster* (Lawrie et al. 2013). Based on these observations, we speculate that the codon usage bias, different evolutionary constraint, among others, may cause the pattern we observed.

**Fig. 3:**
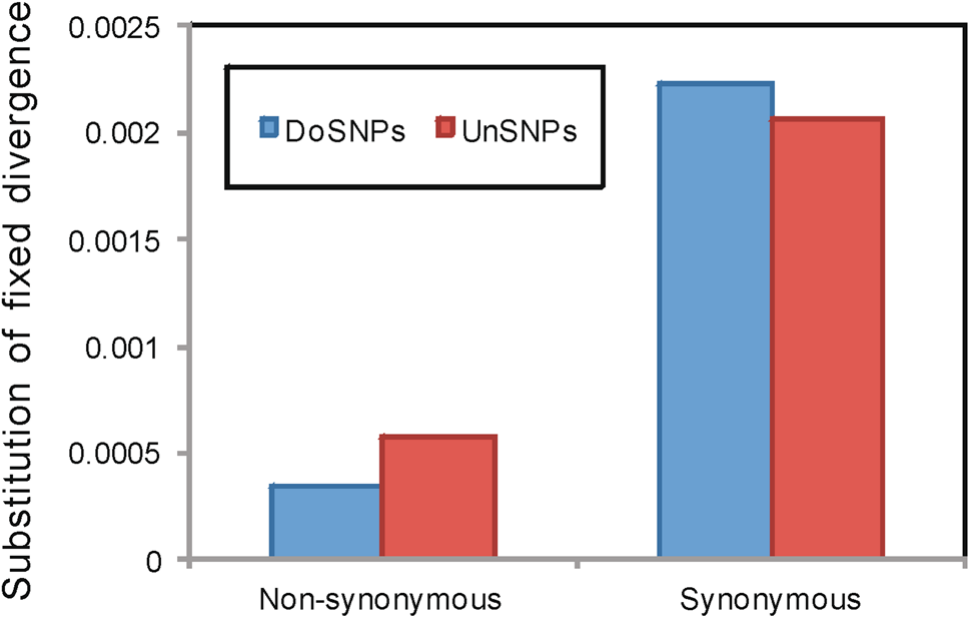
Distribution of non-synonymous and synonymous substitution rates of fixed mutations in domains and unassigned regions

We subsequently surveyed the substitution rates of the fixed mutations (the method is same with the density of SNPs) and found that the patterns were consistent with those of the polymorphisms (Fisher’s exact test: *ρ* = 0.61, *p* < 2.2 × 10^−16^ and *ρ* = 1.08, *p* = 1.6 × 10^−15^, respectively for non-synonymous SNPs and synonymous SNPs) (Fig. 3).

### Domains without SNPs

In our preceding analysis, we found some domains without SNPs, but it was not known whether the SNPs were absent in all transcripts or only in the principal splice isoform. To this end, all annotated transcripts of each protein-coding gene were analyzed. In total, there were 75,795 protein sequences encoded by 20,571 protein-coding genes. Collectively, these protein sequences contained 5464 domains. We found 117 domains with no SNPs (Supplementary Table S1) in this variation dataset. Although they may change when more genomes become available, their rates of substitution are low. Of these, only 25 domains are annotated by “molecular function” of Gene Ontology (Ashburner et al. 2000) (the annotation of domains were downloaded from Pfam27.0) (Table 3).

**Table 3:** Annotation of domains without any variation

Pfam Acc	Average length	Frequency of occurrences	Category ID^a^, category name^b^
PF00220	9	2	GO:0005185, neurohypophyseal hormone activity
PF00416	98.5	2	GO:0003723, RNA binding GO:0003735, structural constituent of ribosome
PF00714	138	1	GO:0005133, interferon-gamma receptor binding
PF00833	122	2	GO:0003735, structural constituent of ribosome
PF01192	53	3	GO:0003899, DNA-directed RNA polymerase activity GO:0003677, DNA binding
PF01200	69	1	GO:0003735, structural constituent of ribosome
PF01472	76.7	3	GO:0003723, RNA binding
PF01648	113	3	GO:0000287, magnesium ion binding GO:0008897, holo-[acyl-carrier-protein] synthase activity
PF01918	65.5	4	GO:0003676, nucleic acid binding
PF02045	57	2	GO:0003700, sequence-specific DNA binding transcription factor activity
PF02229	56	6	GO:0003677, DNA binding GO:0003713, transcription coactivator activity
PF02935	60.7	3	GO:0004129, cytochrome-c oxidase activity
PF02938	97	1	GO:0005524, ATP binding GO:0004812, aminoacyl-tRNA ligase activity
PF03002	18	4	GO:0005179, hormone activity
PF04272	52	1	GO:0042030, ATPase inhibitor activity GO:0005246, calcium channel regulator activity
PF04376	79	5	GO:0004057, arginyltransferase activity
PF05366	31	3	GO:0030234, enzyme regulator activity
PF05495	74	4	GO:0008270, zinc ion binding
PF09282	26.3	3	GO:0005515, protein binding
PF10576	17	3	GO:0051539, 4 iron, 4 sulfur cluster binding GO:0004519, endonuclease activity
PF11411	36	3	GO:0003910, DNA ligase (ATP) activity
PF11547	53	3	GO:0043130, ubiquitin binding
PF11803	46	6	GO:0048040, UDP-glucuronate decarboxylase activity
PF12125	40	9	GO:0046983, protein dimerization activity
PF13014	38	1	GO:0003723, RNA binding

^a^Id of Gene Ontology “molecular function” (from Pfam27.0)

^b^Name of Gene Ontology “molecular function”

To verify the number of domains without any SNPs is statistically significant, we randomly assigned all their N observed SNPs to positions in the human proteins, repeated this random assignment 1000 times (see “Materials and methods”). We obtained two *p* values: the proportion of times that the number of domains without SNPs is greater or equal 117, and the proportion of times that the average of occurrences of domains without SNPs is higher or equal than the one observed for the original 117 domains. Both of them are 0. These indicate that there are significantly greater domains without SNPs than expected at random, and the domains without SNPs are not rare domains.

## Discussion

In the human genome, there are three sources of genome-wide SNP data sets: the Single Nucleotide Polymorphism Database (dbSNPs) (Sherry et al. 2001), HapMap (Altshuler et al. 2010), and the 1000 Genome Project. Half of the reported SNPs in dbSNPs are only candidate SNPs and are not validated in a population (Musumeci et al. 2010). For HapMap, certain genome loci were selected for sequence analysis, so the variations are biased. The 1000 Genome Project reports the genomes of 1092 individuals from 14 populations using whole-genome and exome sequencing. This is a powerful and cost-effective design for discovering variants (Abecasis et al. 2012). Our analysis is based on data from the 1000 Genome Project, which bolsters the accuracy and comprehensiveness of our investigation. Using this data set, we also observed the relationship between the length of protein-coding sequences and variation.

Here, we analyzed 20,571 protein-coding genes, excluding those on the Y chromosome and in the mitochondrial genome. By mapping the SNPs onto the CDSs, only 19,909 genes were found to have variations. To investigate the relationship between the number of SNPs and the length of a protein-coding sequence, we extracted SNPs and the length of each gene. As shown in Table 4, there is a positive correlation between the number of SNPs within a protein and the length of that protein for different MAF SNPs.

**Table 4:** Spearman’s *ρ* and *p* between the number of different MAF SNPs and the length of proteins

SNP categories	Spearman’s *ρ*, *p* of rare MAF SNPs	Spearman’s *ρ*, *p* of low MAF SNPs	Spearman’s *ρ*, *p* of common MAF SNPs
Non-synonymous SNPs	0.83, <2.2 × 10^−16^	0.65, <2.2 × 10^−16^	0.43, <2.2 × 10^−16^
Synonymous SNPs	0.82, <2.2 × 10^−16^	0.70, <2.2 × 10^−16^	0.53, <2.2 × 10^−16^

**Fig. 4:**
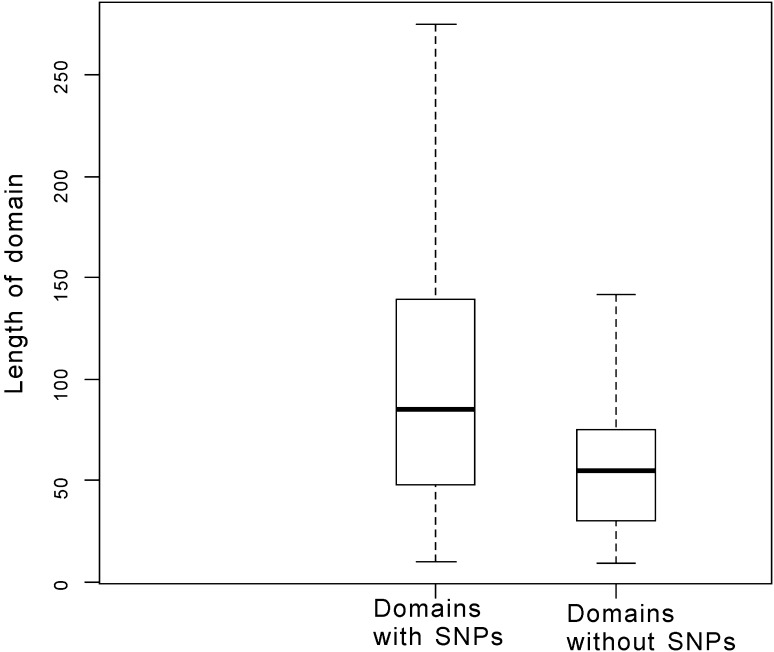
Distribution of domain lengths

However, there remained the question of whether the aforementioned 117 domains are too crucial to tolerate SNPs or too short to have no chance to get SNPs. To answer the question, we analyzed the distribution of domain lengths. Figure 4 illustrates that the median domain length with SNPs was 85, while the median of those without SNPs was 55. This indicated that the two group domains are significantly different in the distribution of lengths (Mann–Whitney *U* test: *p* < 2.2 × 10^−16^). Although the domains without SNPs were short, they were not rare domains (see “Results”). This might increase opportunities for obtaining variations. The average length of the domains without SNPs is 62 amino acids, and the average occurrences of them are 4. The frequency of SNPs is 0.015 (492,826/(14,557,293 + 18,411,513)). For each domain, it would get 2.8 (62 × 3 × 0.015) SNPs on average. But there were significantly more domains without SNPs than expected at random (see “Results”). Therefore, the length may not been the key reason of without SNPs. There are some domains without SNPs are really important. For example, the PF00220 domain is involved in neurohypophyseal hormone activity. It was found that there are two human proteins, encoded by the AVP and OXT genes, respectively, each containing one such domain (residues 20–28). In the 1092 human population dataset, no SNP was recorded in the domain; however, familial neurohypophyseal diabetes has been linked to the mutations occurring within the domain. One unusual familial neurohypophyseal diabetes in Palestine was caused by a missense mutation at nucleotide 77 in the coding sequence encoded by the AVP gene, replacing Pro with Leu (residues 26, CCG → CTG) (Willcutts et al. 1999). This substitution reduced the binding affinity of its host protein to receptors. Another example is familial neurohypophyseal diabetes in Turkey, which was found to be caused by a mutation (T → C at position 61 in coding sequences encoded by the AVP gene). This mutation substituted Try with His (residues 21, TAC → CAC) and led to impaired folding (Rittig et al. 2002). These reports suggest that PF00220 is important for humans.

Synonymous mutations do not alter amino acids and are therefore not considered to alter the function of the protein where they occur. Thus, such mutations have long been thought to lack functional effect or evolutionary importance. Recent research has contradicted this notion (Singh et al. 2007; Weatheritt and Babu 2013). In our studies, we found that synonymous density is less frequent in unassigned regions compared to that in human domains. This may be caused by codon usage bias or different evolutionary constraints between on the synonymous unSNPs and on the synonymous doSNPs.

We must note that our results might be affected by the quality of the datasets upon which our analyses are based. First, in 1000 Genomes pilot data, SNPs have been identified within each population, but allele frequency information are applied to all the populations. Second, although deep (50–100×) exome sequencing strategy was taken in 1000 Genomes project, there are only 1092 individuals and may miss coding sites. Third, the classification of domains and unassigned regions are based on PfamA version 27.0.

In summary, protein evolution is crucial for species evolution. Previous studies have focused on whole proteins, while less attention has been paid to differences within a protein. To our knowledge, this is the first study exploring evolution at the protein domain level within species. The results presented here imply that substitutions in domains and synonymous mutations in other unassigned regions must be taken into consideration for coding sequences. This research may help to further understand human protein evolution and disease.

## Electronic supplementary material

Below is the link to the electronic supplementary material.

Supplementary material 1 (PDF 466 kb)
