# Supplementary material for: Different evolutionary patterns of SNPs between domains and unassigned regions in human protein-coding sequences
Source: Mol Genet Genomics. 2016 Jan 30;291:1127–36. doi: 10.1007/s00438-016-1170-7 (PMC4875946; doi:10.1007/s00438-016-1170-7)
Supplement: Supplementary file 1 — Supplementary material 1 (PDF 466 kb) [file 438_2016_1170_MOESM1_ESM.pdf]

Different evolutionary patterns of SNPs between domains and unassigned regions in the human protein-coding sequences

Erli Pang[1]*, Xiaomei Wu[2], and Kui Lin[1]

Figure legends

Figure S1. Density of SNPs in domains and unassigned regions for the proteins excluded significant difference in amino acid compositions between domains and other unassigned regions.

Figure S2. Density of SNPs in domains and unassigned regions for the proteins excluded codon usage bias.

Table S1. The list of domains without any SNP

Pfam Acc	Average length	Frequency of occurrences	Description
PF00220	9	2	Neurohypophysial hormones, N-terminal Domain
PF00416	98.5	2	Ribosomal protein S13/S18
PF00714	138	1	Interferon gamma
PF00833	122	2	Ribosomal S17
PF01192	53	3	RNA polymerase Rpb6
PF01200	69	1	Ribosomal protein S28e
PF01466	73.9	7	Skp1 family, dimerisation domain
PF01472	76.7	3	PUA domain

PF01541	78.7	6	GIY-YIG catalytic domain
PF01648	113	3	4'-phosphopantetheinyl transferase superfamily
PF01918	65.5	4	Alba
PF02044	13.2	6	Bombesin-like peptide
PF02045	57	2	CCAAT-binding transcription factor (CBF-B/NF-YA) subunit B
PF02229	56	6	Transcriptional Coactivator p15 (PC4)
PF02268	45.2	5	Transcription initiation factor IIA, gamma subunit, helical domain
PF02935	60.7	3	Cytochrome c oxidase subunit VIIc
PF02938	97	1	GAD domain
PF03002	18	4	Somatostatin/Cortistatin family
PF03508	20	2	Gap junction alpha-1 protein (Cx43)
PF03660	106	1	PHF5-like protein
PF03670	83	1	Uncharacterised protein family (UPF0184)
PF03735	71.8	10	ENT domain
PF03823	55	3	Neurokinin B
PF03911	41	1	Sec61beta family
PF04031	122.8	5	Las1-like
PF04063	179	2	Domain of unknown function (DUF383)
PF04064	58	3	Domain of unknown function (DUF384)

PF04272	52	1	Phospholamban
PF04376	79	5	Arginine-tRNA-protein transferase, N terminus
PF04418	63	1	Domain of unknown function (DUF543)
PF05002	71	7	SGS domain
PF05254	71	1	Uncharacterised protein family (UPF0203)
PF05366	31	3	Sarcolipin
PF05391	21	3	Lsm interaction motif
PF05495	74	4	CHY zinc finger
PF06001	42	4	Domain of Unknown Function (DUF902)
PF06345	15	25	DRF Autoregulatory Domain
PF06726	64.7	9	Bladder cancer-related protein BC10
PF06803	41	4	Protein of unknown function (DUF1232)
PF07189	79	1	Splicing factor 3B subunit 10 (SF3b10)
PF07392	51	2	Cyclin-dependent kinase inhibitor 2a p19Arf N-terminus
PF07545	29.1	8	Vestigial/Tondu family
PF07683	101.4	28	Cobalamin synthesis protein cobW C-terminal domain
PF08143	69.3	15	CBFNT (NUC161) domain
PF08145	212.8	4	BOP1NT (NUC169) domain
PF08155	53	3	NOGCT (NUC087) domain

PF08180	28	1	B melanoma antigen family
PF08209	33	2	Sgf11 (transcriptional regulation protein)
PF08288	90	1	PIGA (GPI anchor biosynthesis)
PF08621	49	1	RPAP1-like, N-terminal
PF08799	30	6	pre-mRNA processing factor 4 (PRP4) like
PF08910	99.3	3	Aida N-terminus
PF08914	65	1	Rap1 Myb domain
PF08944	60	2	NADPH oxidase subunit p47Phox, C terminal domain
PF09006	46	1	Lung surfactant protein D coiled-coil trimerisation
PF09050	26	1	Stannin unstructured linker
PF09072	63	1	Translation machinery associated TMA7
PF09282	26.3	3	Mago binding
PF10155	101	2	Uncharacterized conserved protein (DUF2363)
PF10270	93	2	Membrane magnesium transporter
PF10381	25	1	Autophagocytosis associated protein C-terminal
PF10397	85	3	Adenylosuccinate lyase C-terminus
PF10453	58	1	Nuclear fragile X mental retardation-interacting protein 1 (NUFIP1)
PF10461	34	2	Peptidase S68
PF10490	49	1	Rb-binding domain of kinetochore protein Cenp-F/LEK1
PF10562	29	9	Calmodulin-binding domain C0 of NMDA receptor NR1 subunit
PF10571	25	2	Uncharacterised protein family UPF0547
PF10576	17	3	Iron-sulfur binding domain of endonuclease III
PF11027	80.7	3	Protein of unknown function (DUF2615)
PF11404	26	5	Potassium voltage-gated channel
PF11409	41	2	Smad anchor for receptor activation (SARA)
PF11411	36	3	DNA ligase IV
PF11532	30	2	Heterogeneous nuclear ribonucleoprotein M
PF11547	53	3	E3 ubiquitin ligase EDD

PF11587	28	4	Major prion protein bPrPp - N terminal
PF11636	31	1	Troponin I residues 1-32
PF11732	78	4	Transcription- and export-related complex subunit
PF11803	46	6	UDP-glucuronate decarboxylase N-terminal
PF12108	28	1	Splicing factor SF3a60 binding domain
PF12125	40	9	D domain of beta-TrCP
PF12329	74	3	TATA element modulatory factor 1 DNA binding
PF12341	27	3	Protein of unknown function (DUF3639)
PF12491	58	1	Apolipoprotein B100 C terminal
PF12537	70.8	6	Protein of unknown function (DUF3735)
PF12580	194	2	Tripeptidyl peptidase II
PF12832	70	4	MFS_1 like family
PF13014	38	1	KH domain
PF13181	34.5	8	Tetratricopeptide repeat
PF13345	72.8	5	Domain of unknown function (DUF4098)
PF13696	32	1	Zinc knuckle
PF13887	33	4	Myelin gene regulatory factor -C-terminal domain 1
PF13917	40.6	7	Zinc knuckle
PF14304	46.3	4	Transcription termination and cleavage factor C-terminal
PF14369	32	3	zinc-finger
PF14543	142	2	Xylanase inhibitor N-terminal
PF14608	19	18	Zinc finger C-x8-C-x5-C-x3-H type
PF14632	94.5	2	Acidic N-terminal SPT6
PF14641	138	2	Helix-turn-helix DNA-binding domain of SPT6
PF14692	28	1	Domain of unknown function (DUF4462)
PF14773	59	2	Helicase-associated putative binding domain, C-terminal
PF14777	62.3	4	Cilia BBSome complex subunit 10
PF14880	56	1	Cytochrome oxidase c assembly
PF14901	93	4	Cleavage inducing molecular chaperone
PF14952	44	8	Putative treble-clef, zinc-finger,

			Zn-binding
PF14960	51	5	ATP synthase regulation
PF14989	151	2	Coiled-coil domain containing 32
PF15018	38	1	TRP-interacting helix
PF15026	127	2	FAM74 protein
PF15114	62	2	Uncharacterised protein family UPF0640
PF15118	65	1	Domain of unknown function (DUF4560)
PF15125	70	1	TMEM238 protein family
PF15161	63	3	Neuropeptide-like
PF15198	95	1	Dexamethasone-induced
PF15248	65.9	10	Domain of unknown function (DUF4587)
PF15285	25	2	Beclin-1 BH3 domain, Bcl-2-interacting
PF15360	55	1	APJ endogenous ligand
PF15613	34.7	6	WSTF, HB1, Itc1p, MBD9 motif 2

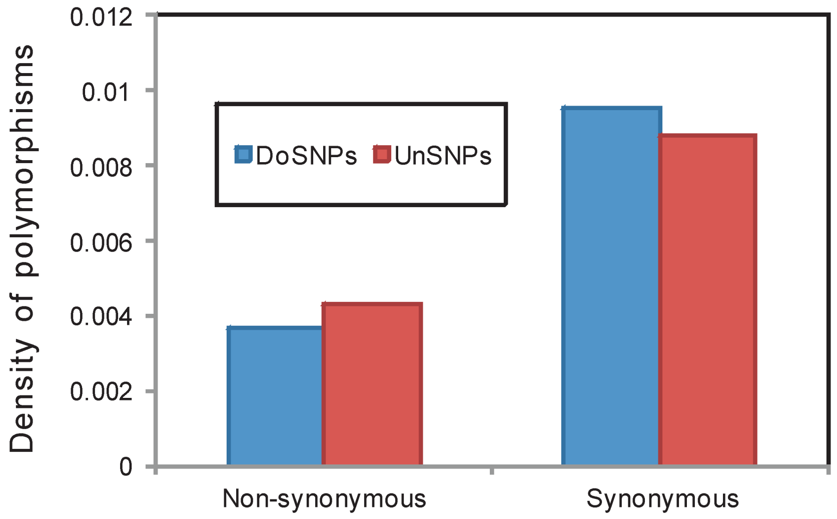
Density of polymorphisms
0.012
0.01
0.008
0.006
0.004
0.002
0
DoSNPs
UnSNPs
Non-synonymous
Synonymous

Figure S1

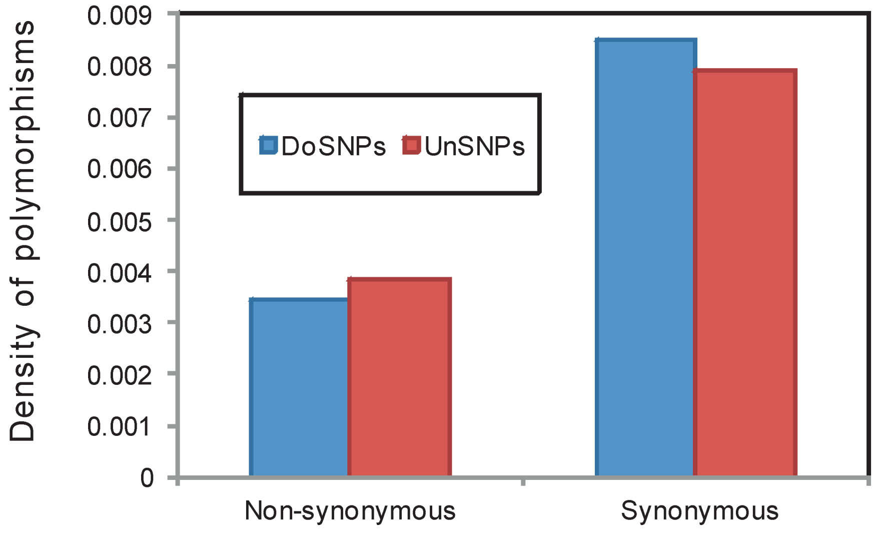
Density of polymorphisms
0.009
0.008
0.007
0.006
0.005
0.004
0.003
0.002
0.001
0
DoSNPs
UnSNPs
Non-synonymous
Synonymous

Figure S2
